# Letter to the editor: COVID-19 cases among school-aged children and school-based measures in Hong Kong, July 2020

**DOI:** 10.2807/1560-7917.ES.2020.25.37.2001671

**Published:** 2020-09-17

**Authors:** MW Fong, BJ Cowling, GM Leung, P Wu

**Affiliations:** 1 World Health Organization Collaborating Centre for Infectious Disease Epidemiology and Control, School of Public Health, Li Ka Shing Faculty of Medicine, The University of Hong Kong, Hong Kong Special Administrative Region, China

**Keywords:** COVID-19, school, children, measures, interventions

**To the editor:** We read with interest the recent rapid communication by Stein-Zamir et al. analysing a major outbreak in an Israeli high school, which has been attributed to crowded conditions in classrooms and exemption from wearing face masks [1]. We would like to share our perspective from Hong Kong, where cases among school-aged children have been reported but did not lead to school outbreaks.

As part of the response to coronavirus disease 2019 (COVID-19), schools in Hong Kong did not resume after the Lunar New Year holiday at the end of January 2020. Classes were instead scheduled online. Following a period without any local infections, secondary schools reopened in late May and primary schools reopened in the subsequent weeks. There were no cases in school-aged children until early July when local transmission resurged [2]. Schools were closed again on 13 July, 1 week before the scheduled summer break. By 18 July, there were 20 cases aged 5–17 years. Fifteen were linked to case clusters within their own household or neighbourhood or had unknown source of infection. The remaining cases included a secondary school cluster and a cluster at a tutorial centre.

Assuming that students were potentially infectious from 4 days before illness onset through 7 days after onset [3], many cases attended school while infectious (Figure). School-wide testing was conducted for schools attended by seven of the 15 cases and for the two clusters, and close contacts were placed under medical surveillance. No other cases related to these 20 cases have been identified in this age group since, suggesting that multiple potential introductions of COVID-19 into schools did not lead to onward transmission. This may be because children, especially young ones, could be less efficient spreaders of COVID-19 [4,5], supplemented by the protective effect of school-based precautionary measures.

**Figure:**
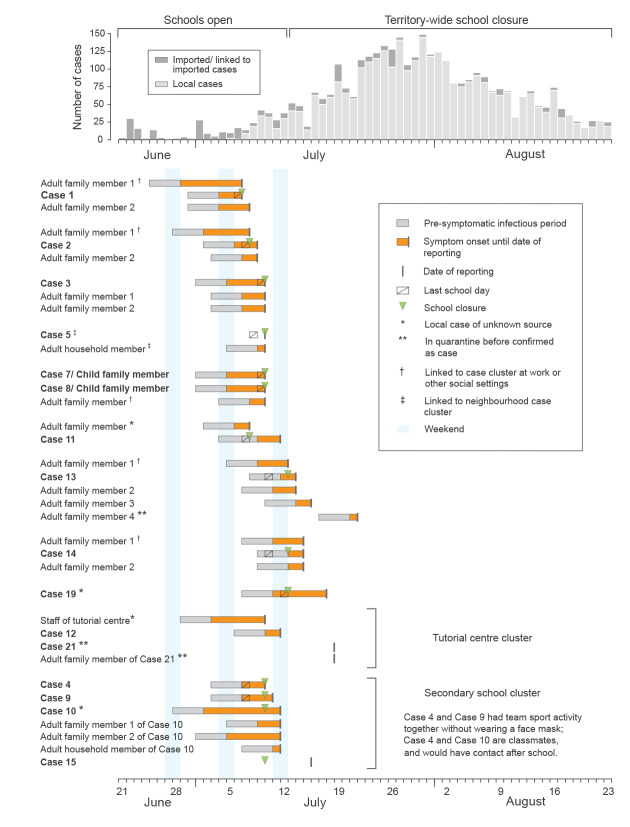
School-aged COVID-19 cases identified during school resumption (27 May–12 July) until the 1st week of territory-wide school closure (13–18 July), Hong Kong

Top panel: epidemic curve of daily case numbers from June through August in Hong Kong, with cases shown by date of reporting. Lower panel: information on individual cases by cluster. Cases with no information on school attendance and not linked to other school-aged cases (Cases 16, 17, 18 and 20) are not shown in this figure. Case 6, an asymptomatic case linked only to neighbourhood case cluster is not shown here either. Case 21 was reported after 18 July but included in this figure because the case was linked to the tutorial centre cluster. The pre-symptomatic infectious period was assumed to begin 4 days before symptom onset [3]. Cases were typically isolated on the day of reporting or up to 2 days before reporting, ending any possible transmission. Schools for Cases 1–3, 5, 7–8, 11 and the secondary cluster were closed before the territory-wide school closure, while Cases 13, 14 and 19 were reported after 10 July, therefore these schools were closed with the territory-wide school closure.

Various infection control measures were adopted by local schools during the school resumption. Staff and students underwent daily temperature checks upon arrival at school. Face masks were worn at all times, and schools switched from full-day to half-day mode omitting lunch hours. Students’ arrival and dismissal times were staggered or spread out using multiple entrances, desks in classroom were spaced out, and some schools installed transparent partitions between desks. Group work and contact sports were limited as much as possible. To avoid mixing of students from different classes and grades, assemblies, extra-curricular and after-school activities were cancelled, and usage of common facilities was staggered. More efforts to ensure distancing between staff and students will further improve the current strategy in view of the higher infection risk among adults [6]. Previous responses from local schools varied from flexible attendance policies and immediate dismissal to closure for varying durations; this indicated an urgent need to have standardised preparedness plans containing measures to be taken by schools in response to confirmation of cases or contacts of a COVID-19 case among staff and/or students.
